# The attitudes of international medical students toward educational methods and styles applied in a 6-year longitudinal course in fundamentals of medical skills in Croatia

**DOI:** 10.3325/cmj.2018.59.267

**Published:** 2018-10

**Authors:** Ines Potočnjak, Monika Elisabeth Crumbach, Anna Mara Hrgetić Vitols, Sandra Hrnčić, Christopher Lambers, Marijana Braš, Davor Ježek, Sven Seiwerth, Vesna Degoricija

**Affiliations:** 1 Department of Medicine, *Sestre Milosrdnice* University Hospital Center, Zagreb, Croatia; 2 Medical Studies in English, University of Zagreb School of Medicine, Zagreb, Croatia; 3 Pediatric polyclinic dr. Diana Puževski, Zagreb, Croatia; 4 University of Zagreb School of Medicine, Zagreb, Croatia; 5 Department of Psychiatry, University Hospital Center Zagreb, Zagreb, Croatia; 6 Department of Histology and Embryology, University of Zagreb School of Medicine, Zagreb, Croatia; 7 Clinical Department of Pathology and Cytology, University Hospital Center Zagreb, Zagreb, Croatia; # Members of Student council

## Abstract

**Aim:**

To investigate international medical students’ attitudes toward the impact of 6-year longitudinal course, Fundamentals of Medical Skills (FMS), at Medical Studies in English at the University of Zagreb on the development of their practical, clinical, and communication skills.

**Methods:**

This cross-sectional study used a 23-item online survey to collect data from five generations of students attending the FMS course from January 31 to February 3, 2017. First-year students were not included. Invitations and reminders were sent to 202 FMS students by e-mail, SMS, and in closed groups in social networks

**Results:**

The response rate was 69.8% (141/202 students). The majority of students found the course useful (83.7%); favored practical over communication skills (92.9%); found practical skills more useful in higher years (82.3%); thought more time was needed to practice by simulation on mannequins (75.2%); preferred Objective Structured Clinical Examination (OSCE) stations to traditional oral exams (78%); and would recommend a course like FMS to future students or students at other universities (79.4%). Significantly more women than men favored practical over communication skills (*P* = 0.044). Significantly more 5th and 6th students than students at lower years preferred OSCE stations to traditional learning (*P* = 0.025) and would recommend a course like FMS to future students or students at other universities (*P* = 0.001).

**Conclusion:**

Students positively evaluated the FMS course, but underestimated the communication skills aspect.

Setting standards for undergraduate medical education is a crucial but still developing research area in Croatia (1). Improvement and innovation in teaching and education are primary goals of the University of Zagreb Medical Studies in English (MSE) program. As part of this program, the 6-year longitudinal course Fundamentals of Medical Skills (FMS) was introduced in 2011, with English as the official language. The course focuses on the development of communication (2-4), practical, and clinical skills with an aim to prepare the students for residency or clinical practice. The content follows the core curriculum subjects and combines essential knowledge, skills, and algorithms of various clinical disciplines (washing hands, basic, advanced, pediatric and trauma life support, drawing blood, urinary catheter insertion, nasogastric tube insertion, intravenous/intramuscular/subcutaneous injection, out-of-hospital labor, immobilization, clinical case simulations, role-playing situations, standardized patients). The content is delivered through simulation on mannequins, role-play, and meet-my-patient scenarios.

FMS has adopted the Calgary-Cambridge model (5), emphasizing students’ active role in the educational process (6). Also, it meets the current standards by teaching physical examination in an appropriate longitudinal curriculum (7,8) and teaching core competences in undergraduate programs (9). In order to evaluate the training success, appropriate assessments of clinical skills are implemented (10).

In the academic year 2016/2017, the course involved 15 tutors and 202 students. The same tutor follows a group of 10 students throughout all six years, for 30 teaching hours per each academic year. Tutors/mentors should have basic core teaching skills (11), be trained for their role (11-14), and provide high-quality education (15). Teaching is standardized through a two-day course for tutors, lesson plans, and a tutors’ guidebook. End of year exam comprises three Objective Structured Clinical Examination (OSCE) stations and a communication skills essay (16,17). Simulation, used in OSCE stations, is useful in teaching skills, and developing self-confidence in a safe environment without the possibility of causing real harm (18,19).

It is not known how clinical, practical, and communication skills training improves students’ abilities. Although it is important to assess students’ attitudes toward a particular course, this feedback is seldom reported (20). Students usually get feedback about their performance (21), but here we investigated students’ opinions about the course and teaching styles. The aim of this research was to investigate the international medical students’ attitudes toward the 6-year longitudinal course on development of their practical, clinical, and communication skills. Our hypothesis was that international medical students thought that the course had a positive impact on the development of their practical, clinical, and communicational skills.

## MATERIALS AND METHODS

### Design

This cross-sectional study included students attending the FMS course. The research was carried out as an online survey among five generations of students (2nd-6th year) from January 31 to February 3, 2017. Invitations and reminders were sent to students by e-mail, SMS, and in closed groups in social networks. Reminders were sent once after two days. Only FMS students were invited. Respondents’ anonymity was protected by analyzing all collected data; no identification data were collected.

### Participants

A total of 202 students were invited to participate, 141 of whom (69.8%) responded. Involved participants were from Austria (n = 1), Bosnia and Herzegovina (BiH) (n = 2), Cameron (n = 1), Canada (n = 7), Croatia (n = 35), United Kingdom (n = 2), Finland (n = 1), France (n = 10), Germany (n = 12), Israel (n = 22), Mauritius (n = 1), Morocco (n = 1), Norway (n = 1), Pakistan (n = 1), Peru (n = 1), Slovenia (n = 8), South Africa (n = 1), Spain (n = 2), Sweden (n = 18), United States of America (USA) (n = 7), Venezuela (n = 1), and other countries (as stated by students) (n = 6).

### Materials

The questionnaire, designed in Google Forms (FMS Evaluation form, Supplementary material(supplementary questionnaire)), collects data on age, gender, country of origin, study year, and FMS-related information. It comprises 23 questions: four open-ended, eight single-choice, one multiple-choice, seven yes/no, and three 5-point Likert scale questions. Only invited participants were able to respond.

### Measures

The responders were divided into 3 groups based on their age – 19-21, 22-26, and 27-35 years and in two groups based on their country of origin – Regional (Croatia, BiH, and Slovenia) and Foreign (all other countries). The latter division was based on language and cultural similarities between the countries. The questionnaire items were rated on a 5-point Likert scale from 1 = strongly disagree to 5 = strongly agree. The following tutor/mentor characteristics were identified: communication, approachability, friendliness, knowledge, flexibility, English skills, leadership, and other.

### Statistical analysis

Descriptive analysis of the responses was performed. Normality of the data distribution was not tested since all the variables were discrete and their distribution is given with frequency tables. The independence of two variables was tested using Fisher exact test instead of Pearson χ^2^ if there was an expected frequency of fewer than 5 in one or more cells of the contingency table. *P* < 0.05 was considered significant. Statistical analysis was performed using IBM SPSS Statistics – trial version (*https://www.ibm.com/analytics/spss-trials*), June 2017.

## RESULTS

### Descriptive analysis

The response rate was 69.8% (141/202 students). There were more female respondents (Table 1). One hundred and eighteen (83.7%) students found the course useful. One hundred and thirty-one students (92.9%) favored practical and clinical skills over communication skills, while only 16 (11.3%) thought more focus should be put on communication skills. As many as 122 (86.5%) students preferred the method of teaching using real patients, and 88 (62.4%) were able to use the skills acquired through FMS in other courses. One hundred and sixteen (82.3%) students found practical skills more useful in the higher years, and 106 (75.2%) thought that more time was needed for simulation on mannequins. Additionally, 40 (28.4%) students strongly agreed (Likert scale 5), 30 (21.3%) agreed (Likert scale 4), 39 (27.7%) did not agree or disagree (Likert scale 3), 17 (12.1%) disagreed (Likert scale 2), while 15 (10.6%) students strongly disagreed (Likert scale 1) that during their first years FMS made them feel closer to practical and clinical aspects of medicine. When asked how they valued having FMS throughout their studies, 75 (53.2%) students found it was useful to review every year, 38 (27.0%) thought they needed this approach in more classes, and 15 (10.6%) claimed to have forgotten all the knowledge between the years. Also, 110 (78%) students preferred OSCE stations, 8 (5.7%) students preferred traditional oral exams, whereas 23 (16.3%) students did not know which they preferred. Fifty-two (36.9%) students graded their teachers with 5 (excellent), 54 (38.3%) with 4 (very good), 25 (17.7%) with 3 (good), 5 (3.5%) with 2 (satisfying), and 5 (3.5%) with 1 (unsatisfying). Among teachers’ qualities, knowledge (89.4%), communication skills (75.2%), and approachability (73%) were most valued, followed by friendliness (56.7%), English skills (56%), flexibility (46.8%), leadership (34.8%), and other (9.2%). Additionally, 100 (70.9%) students would participate in other years’ practical exercises either as student demonstrators or volunteers for the OSCE station. Hundred and twelve (79.4%) students would recommend a course like FMS to future students or students at other universities. There was no significant difference in answers to any of the questions among different age groups. On open ended questions students answered that they valued more practical skills and wanted more practice of practical and clinical skills.

**Table 1:** Demographic characteristics of 141 respondents

Characteristics	No. (%) of students
**Age groups (years)**	
19-21	26 (18.4)
22-26	86 (61)
27-35	29 (20.6)
**Gender**	
women	76 (53.9)
men	65 (46.1)
**Country of origin**	
Regional (Croatia, Bosnia and Herzegovina, Slovenia)	45 (31.9)
Foreign (all other countries)	96 (68.1)
**Year of study**	
2nd	26 (18.4)
3rd	33 (23.4)
4th	25 (17.7)
5th	32 (22.7)
6th	25 (17.7)

### Difference according to gender

Significantly more women than men found practical and clinical skills more useful than communication skills (χ2 = 5.208, df = 1, *P* = 0.044), and thought that the FMS course did not require a written exam (χ2 = 8.971, df = 2, *P* = 0.009). Significantly more men than women preferred OSCE stations to traditional learning (χ2 = 9.020, df = 2, *P* = 0.010). There was no significant difference in answers to other questions.

### Difference according to the country of origin

Significantly more foreign than regional students found practical and clinical skills more useful than communication skills (χ2 = 7.185, df = 1, *P* = 0.012), believed that English language quality (χ2 = 5.114, df = 1, *P* = 0.029) and leadership quality were important characteristics of their FMS teacher (χ2 = 4.576, df = 1, *P* = 0.038), and wanted more practice time on mannequins (χ2 = 6.154, df = 2, *P* = 0.045). There was no significant difference in answers to other questions.

### Difference according to the study year

**Figure 1:**
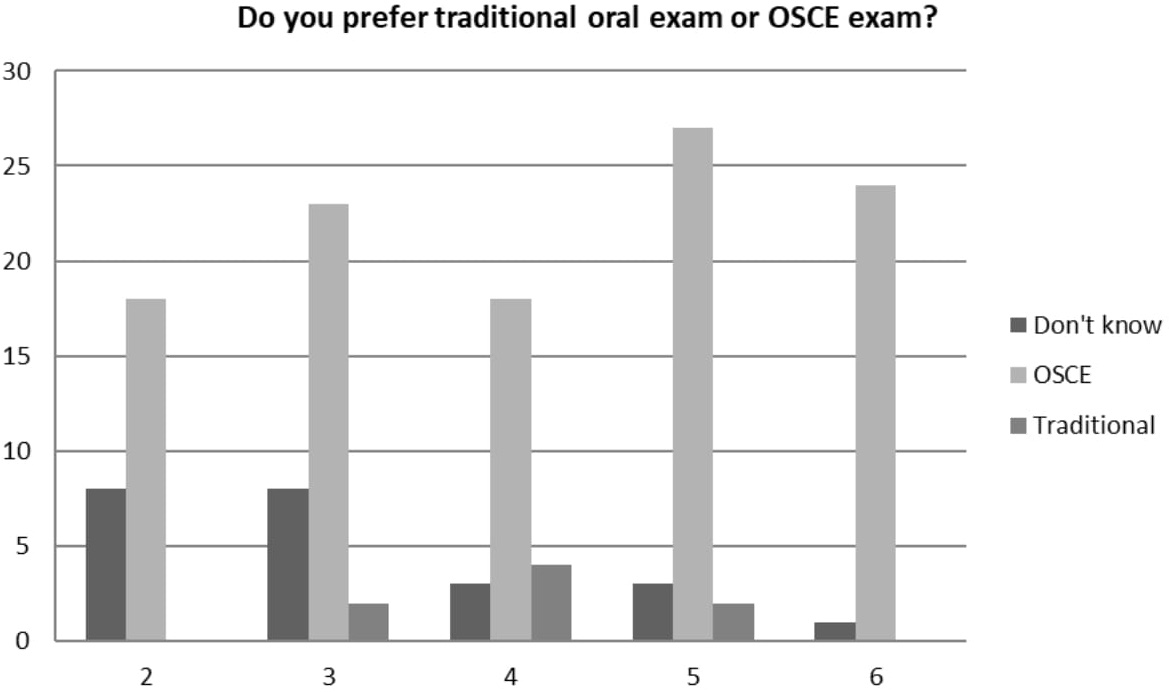
Differences among students of different study years in their preference for traditional oral exam vs Objective Structured Clinical Examination (OSCE) exam. Senior-year students more often preferred OSCE stations (χ2 = 15.303, df = 8, *P* = 0.025).

**Figure 2:**
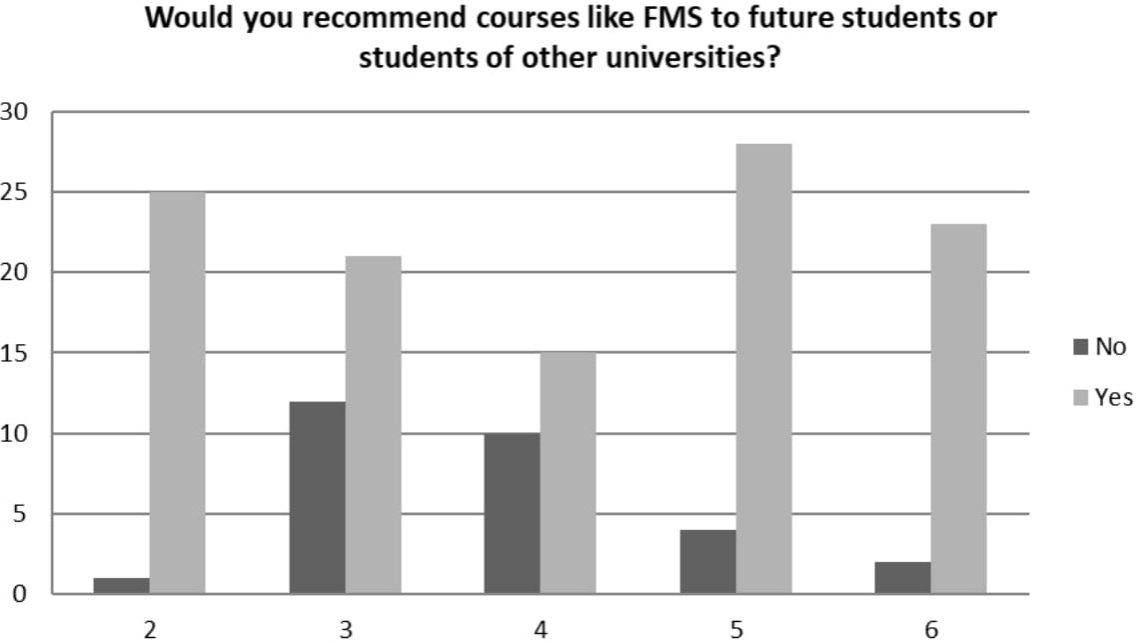
Differences among students of different study years in their willingness to recommend courses such as Fundamentals of Medical Skills (FMS) to future students or students at other universities. Senior-year students would more often recommend courses like FMS to future students or students at other universities (χ2 = 18.960, df = 4, *P* = 0.001).

**Figure 3:**
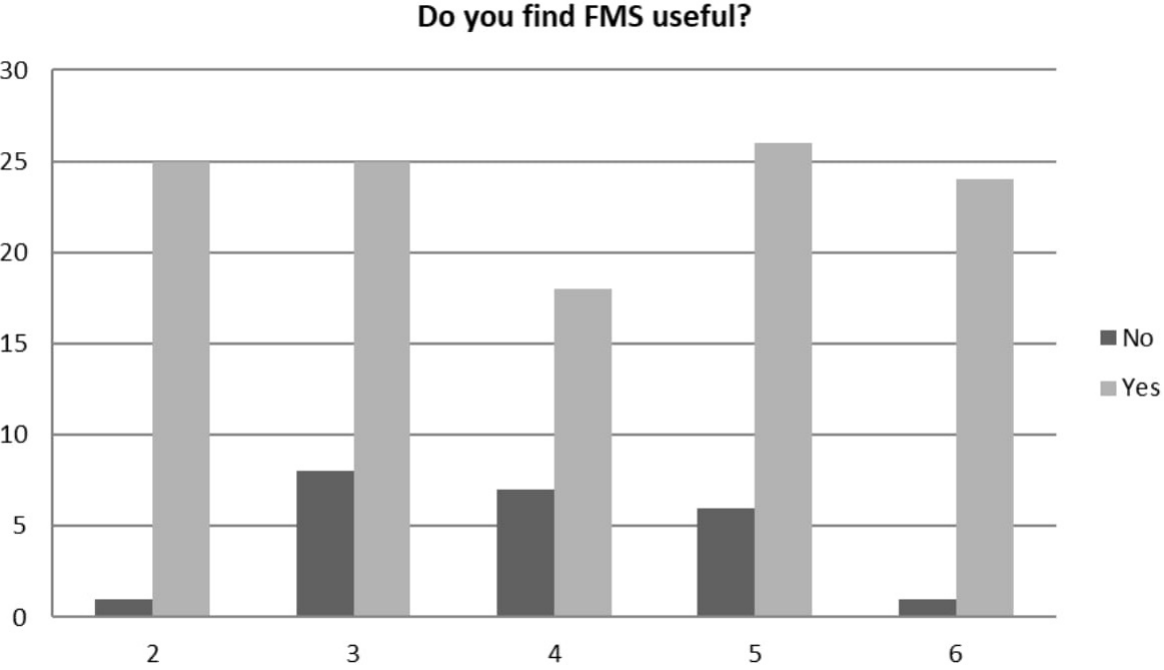
Differences among students of different study years in their attitude towards the usefulness of Fundamentals of Medical Skills (FMS). Second- and sixth-year students found FMS more useful than other students (χ2 = 10.091, df = 4, *P* = 0.032).

There was a significant difference in answers among students of different study years (χ2 = 13.018, df = 8, *P* = 0.033). Significantly more senior year students compared with students at lower years preferred OSCE stations to traditional learning (χ2 = 15.303, df = 8, *P* = 0.025) (Figure 1) and would recommend courses like FMS to future students or students at other universities (χ2 = 18.960, df = 4, *P* = 0.001) (Figure 2). Significantly more second- and sixth-year students found FMS useful than students from other years (χ2 = 10.091, df = 4, *P* = 0.032) (Figure 3). There was no significant difference in answers to other questions.

## DISCUSSION

Our main finding was that students found a positive impact of the FMS course on their clinical and practical skills, but they underestimated the importance of communication skills. As expected, they found practical skills more useful in the higher years and implemented the skills acquired through FMS in other courses. Insufficient recognition of communication skills is in contrast to the findings of Tanwani (2), who reported that students considered training in communication skills laboratory useful. The attitude of our students reflects a possible limited and less critical approach to medical profession, which necessitates excellent social skills for work with patients. The communication skills taught within FMS should be reinforced and students continuously mentored in role-play scenarios and work with real patients. Since communication skills are imperative in clinical work, their development is essential in high-quality undergraduate training (3,22). An important way to achieve this is to provide students with adequate feedback (21).

It was expected that students would find practice on patients more valuable than that on mannequins and simulators because clinical experience is very valuable for their future work. However, they wanted more time for practice on simulation mannequins.

Senior-year students more often preferred exams on OSCE stations and would more often recommend courses like FMS to future students or students at other universities. It is possible that taking clinical courses made them aware of the importance of practical and communication skills. Second- and sixth-year students found FMS more useful than other students, which can be explained by the fact that sixth-year students realize the importance of skills in their work. Male students preferred OSCE station exams less. Interestingly, previous studies showed that female students performed better in communication than male despite rating their communication skills lower (4). In our study, women found practical skills more useful. Furthermore, significant behavioral differences between female and male doctors were found (23), which might be balanced during undergraduate study. Bertakis (23) found that female doctors provided more preventive services and psychosocial counseling. Perhaps women communicate more effectively than men, which might be why our female students found practical skills more useful than communications skills. However, the inability to provide quality consultations to patients might lead to obtaining flawed information (24). Nevertheless, students often overestimate or underestimate their communication skills (25).

Foreign students found the English language skills and leadership quality of the FMS mentors more important than students from the region, who understand the language. This is an interesting contrast to their attitude that practical skills were more important than communication skills. Lack of language skills among foreign students may limit their patient interaction in contrast to regional students, which may lead to underestimating the importance of communication skills. The longitudinal approach during all six years enables students to gradually develop skills and their mentors to follow up, observe improvement, and enhance student development by the individual approach in small groups. The primary outcome is to teach medical students practical and communication skills in clinical disciplines, which they will apply in their fields of work. In our opinion, communication as well as practical and clinical skills are best taught in a longitudinal course such as FMS. We wish to emphasize the importance of challenging traditional methods of medical education. Delivery of the FMS course by a multidisciplinary team of appropriately trained tutors provides diversity in the quality of teaching. Acquiring adequate communication skills is crucial in undergraduate medical education. Alongside practical and clinical skills, communication skills are an integral part of the longitudinal course FMS within the University of Zagreb MSE program.

The limitation of the study was its cross-sectional design using a voluntary online survey, without the use of a randomized design. There was no control group. The questionnaire answers provided also might have limited students in their responses.

The longitudinal approach of the FMS course during all six years enables students to gradually develop skills and allows their mentors to follow up, observe improvement, and enhance student development using individual approach in small groups. In our opinion, communication as well as practical and clinical skills are best taught in a longitudinal course such as FMS. We wish to emphasize the importance of challenging traditional lecturing methods of medical education. Delivery of the FMS course by a multidisciplinary team of appropriately trained tutors provides diversity in the quality of teaching.

To conclude, the international medical students found that FMS course generally positively affected the development of their practical, clinical, and communicational skills. However, the communication skills part of the training was underestimated. This problem can now be addressed, resolved, and continuously improved. The same group of students should be tested when they enter the clinical practice to assess if their attitude toward communication skills would change.

## Additional Material

Supplementary questionnaire
