# Supplementary material for: The attitudes of international medical students toward educational methods and styles applied in a 6-year longitudinal course in fundamentals of medical skills in Croatia
Source: Croat Med J. 2018 Oct;59(5):267–73. doi: 10.3325/cmj.2018.59.267 (PMC6240827; doi:10.3325/cmj.2018.59.267)
Supplement: Supplementary questionnaire [file CroatMedJ_59_s007.pdf]

FMS EVALUATION FORM

This form serves as an evaluation for the course of FMS in its entirety. The survey is anonymous and the data will be used for research purpose and for improvement of the course. Please take a few minutes to fill out the form to the best if your knowledge.
We appreciate your cooperation.

MSE student council

1. What is your age? *

____

2. What is your gender? *

- ◯ Male
- ◯ Female

3. Which country do you come from? *

____

4. Which year are you in? *

- ◯ 1
- ◯ 2
- ◯ 3
- ◯ 4
- ◯ 5
- ◯ 6

5. Do you find practical and clinical skills or communication skills more useful? *

- ◯ Practical
- ◯ Communication

6. Would you say the course of FMS requires a written exam? *

- ◯ Yes
- ◯ No
- ◯ Maybe

7. Which position did your FMS teacher work as during the course? *

- Professor
- Assistant
- Specialist
- Resident
- I don't know

8. How would you grade your FMS teacher? (1-bad; 5-excellent) *

1	2	3	4	5
◯	◯	◯	◯	◯

9. Which qualities do you look for in your FMS mentor? (Select multiple) *

- [ ] Communication
- [ ] Approachability
- [ ] Friendliness
- [ ] Knowledge
- [ ] Flexibility
- [ ] English skills
- [ ] Leadership
- [ ] Ostalo: ____________

10. Do you think practical skills are more useful in higher years? *

- Yes
- No

11. Do you prefer traditional learning (lectures, seminars, practicals) or OSCE stations? *

- Traditional
- OSCE
- Don't know

12. Do you prefer learning on mannequins or live patients? *

- Mannequins
- Live patients

13. Would you find it useful to participate in other year's practicals as either a student demonstrator or a volunteer for the OSCE type of station? *

- Yes
- No

14. Would you recommend courses like FMS to future students or students to other universities? *

- Yes
- No

15. Have you been able to utilise the skills you've acquired in other courses during your study? *

- Yes
- No

16. During your first years did FMS make you feel closer to the aspects of practical and clinical medicine? (1-disagree; 5-agree) *

1	2	3	4	5
◯	◯	◯	◯	◯

17. What would you improve in the FMS course? *

18. How did you value the longitudinal approach of having FMS throughout all years? *

- We need this approach in more classes
- I forgot all the knowledge between the years
- It was useful to review every year
- Ostalo: ____

19. Do you find FMS useful? *

- Yes
- No

20. Evaluate if your clinical skills and practical methods improved. (1-bad; -excellent) *

1	2	3	4	5
◯	◯	◯	◯	◯

21. Do you think more practical time is needed? *

◯ Agree
◯ Disagree
◯ Don't know

22. Would you think the course requires more focus on communication skills? *

◯ Yes
◯ No
◯ Maybe

23. What would you do to improve FMS course quality? *

____
____
____
____
____
